# Parenting During the COVID-19 Pandemic

**DOI:** 10.31729/jnma.5319

**Published:** 2020-11-30

**Authors:** Utkarsh Karki, Gunjan Dhonju, Arun Raj Kunwar

**Affiliations:** 1 Child and Adolescent Psychiatrist, Child and Adolescent Psychiatry Unit, Kanti Children's Hospital, Kathmandu, Nepal

**Keywords:** *COVID-19*, *parenting*, *pandemic*

## Abstract

Parenting is both an art and science of nurturing a child that comes very naturally to some. In today's world, the fundamentals of parenting are getting challenged but it has been even more magnified during the coronavirus 2019 (COVID-19) pandemic. The mental health of children is bound to be affected by the stress related to COVID-19 owing to loss of usual routine, unpredictability, uncertainty. Various other social, economic, and environmental factors also threaten their mental well-being. Parents are the child's first and longest-lasting context for development. Positive parenting that involves sensitivity, responsivity, caring, communicating, and empowering would ensure positive developmental outcomes in children and adolescents. Positive parenting and self-care of parents would serve as a promotive and preventative intervention for child and adolescent mental health, especially during this crisis.

## INTRODUCTION

Parenting is the task of upbringing a child by stimulating the emotional, intellectual, physical, and social development from infancy to adulthood.^1^ It influences a child's mental health because it mediates child-environment interactions and moldsa child's adaptation.^2^ COVID-19 has been affecting children, adolescents, and their families in an unprecedented manner. There is more harm than benefit due to COVID-19 especially for children and hence parenting becomes even more paramount when children are deprived of other opportunities for their personal growth and development.

## PARENTING

Parenting is derived from the Latin verb “parere” which means to bring forth, develop, or educate.^3^ Ecological systems theory views the child as developing within a complex system of relationships and contextual influences affected by multiple levels of the surrounding environment.^4^ The nested structures of the microsystem, mesosystem, exosystem, and macrosystems join with each other to powerfully affect child development. In this system, the parents or immediate family is the child's first, and longest-lasting, context for development. Thus, it is crucial that we focus primarily on parents or immediate family with whom children are in close contact for the majority of the time and more so during this COVID-19 pandemic.

Diana Baumrind initially identified three different types of parenting styles: authoritative, authoritarian, and permissive parenting.^5^ This parenting style is categorized based on two dimensions of parenting behavior i.e. demandingness and responsiveness. Later in 1983 Maccoby and Martin expanded Baumrind's permissive parenting style into two different types: permissive parenting and neglectful parenting.^6^ Authoritative parenting style with high demandingness and high responsiveness is considered the best parenting style.

## PARENTING AND ITS CHALLENGES DURING THE COVID-19

For children, the pandemic has resulted in the closure of schools, limited contacts with peers and family members, restriction to outdoor leisure activities, and difficulty accessing mental health services. Parents have been equally affected adversely with mental health problems, loss of jobs, loss of loved ones, and increased responsibility of working from home and supporting their children at home.^7^

The combination of difficulty in engaging with children, keeping them safe at home,^8^ and significant stress for several parents all over the world^9^ is the major challenge in most of the households. The consequence of social distancing, lockdown, and refrained outdoor activities have resulted in many children being anxious, bored, sad, aggressive, and affecting the emotions and future aspirations of adolescence.^10^ Studies conducted in China in children and adolescents have found clinginess, irritability, fear,^11^ and symptoms of depression and anxiety in approximately 20 percent for each.^12^ Boredom, ease of access, and availability of gadgets leading to unhealthy use of technology has been another major issue for both children and parents.

## POSITIVE PARENTING DURING THE PANDEMIC

Parents should provide a conducive environment at their home to ensure children's mental well-being and healthy developmental trajectory. It is normal for parents to feel overwhelmed during this pandemic but steps to carry out positive parenting with children during this time could benefit both.

Reassure children with facts and let them know that parents are there to keep them safe. It can be taken as an opportunity for parents to empower children to learn values of caring for others, thinking about their community, and educating them about good habits like hand washing, using masks, and covering one's cough. Parents must maintain their calm. Self-care is a priority for parents is important for managing their stress and subsequently their children's stress. Give information to children in an age-appropriate manner about the pandemic and challenges it brings to their life. Parents could keep their children engaged in board games, watching movies, singing, painting, reading a storybook, cooking meals together, activities such as arts, crafts, and indoor stretches to stay active. Parents must allow children to ask questions, discuss their feelings about the pandemic and how it affects them. Parents need not always have an answer and should refrain from false promises but simply being present to listen can go a long way.^13^ Lastly, healthy use of technology, keeping children hydrated, healthy eating habits, maintaining a routine with a time-table, and promoting physical activity benefits children's mental and physical well-being.

Adolescents may have some age-specific issues, which need to be taken into consideration by parents. They will have a better understanding of the COVID-19 related issues compared to children. Parents should keenly observe for any emotional or behavioral changes in their adolescent kids. Parents can play a vital role in ensuring that adolescents maintain their mental health by listening to them, acknowledging their difficulties, clarifying their doubts, reassuring them, generating hope, and providing emotional support in resolving issues.^14^ Excessive use of gadgets can result in behavioral addiction. Parents have to negotiate with adolescents to ensure the limited use of gadgets and to discuss the inclusion of healthy gadget-free activities as a part of the daily routine. Urgent professional help has to be sought if the behavioral and/or emotional changes are pervasive, persistent, impair the functioning of the adolescent, if there is physical aggression towards others, if the adolescent expresses suicidal ideas or hopelessness or attempts self-harm, and in case of any use of alcohol or other substances.

Striving to ensure appropriate care of children, parents may tend to neglect their well-being. Parents should master to strike a balance between their job, household chores, and quality time with children. Furthermore, there is a great need for parents to emphasize their well-being focusing on healthy nutrition, physical exercise, adequate rest, and sleep. On a positive note, the current crisis can serve as an opportunity for personal growth, family cohesion, and to allow a healthy parent-child relationship.

## WAYS FORWARD

COVID-19 is a massive problem all around the globe it is a challenging time for children and parents. However, parents have a significant role to play that could succor children's mental well-being and healthy development. Positive parenting could assist children to oversee the impact of this pandemic by fostering resilience. It can also ensure better development outcomes in behavior, intellectual, emotional, and social skills. This is a difficult time for all with no easy answers but some of these simple strategies may benefit children and parents.
